# Efavirenz induces autophagy and aberrant differentiation in normal human keratinocytes

**DOI:** 10.3892/ijmm.2013.1327

**Published:** 2013-04-03

**Authors:** QINGHUA DONG, JU-EUN OH, JIN KYU YI, REUBEN H. KIM, KI-HYUK SHIN, RONALD MITSUYASU, NO-HEE PARK, MO K. KANG

**Affiliations:** 1 School of Dentistry, University of California, Los Angeles (UCLA), Los Angeles, CA, USA; 2 Jonsson Comprehensive Cancer Center, University of California, Los Angeles (UCLA), Los Angeles, CA, USA; 3 David Geffen School of Medicine, University of California, Los Angeles (UCLA), Los Angeles, CA, USA

**Keywords:** efavirenz, autophagy, keratinocytes, differentiation, p53

## Abstract

Although efavirenz (EFV) is efficacious as an anti-retroviral therapy when combined with other antiretroviral drugs, it may cause adverse clinical effects, including skin and mucosal eruptions, central nervous system complications, hepatotoxicity, renal failure and pulmonary complications. The present study investigated the phenotypic alterations caused by EFV in normal human keratinocytes (NHKs) and determined the cell death pathways leading to the lack of epithelial proliferation and regeneration. Replication kinetics, cellular morphology, and protein and mRNA levels of cell cycle regulatory genes and cell death markers were compared between the EFV-exposed cells and the untreated control. EFV treatment led to cell proliferation arrest and cell death of the NHKs by inducing autophagy mediated by proteasome-dependent degradation of p53. EFV also reduced the levels of mTOR and active ERK signaling in NHKs. Chemical inhibition of p53 degradation with a proteasome inhibitor led to reduced autophagic response of NHKs to EFV. In addition, EFV triggered terminal differentiation of NHKs by inducing the expression of involucrin, filaggrin, loricrin and genes involved in cornified envelope formation. Inhibition of autophagy in the EFV-treated NHKs with 3-methylalanine reduced the levels of involucrin and the extent of cell death. Our data indicate that EFV elicits cytotoxic effects on NHKs in part through induction of autophagy and aberrant differentiation of cells.

## Introduction

While highly active antiretroviral therapy (HAART) reduces the morbidity and mortality of patients infected with the human immunodeficiency virus (HIV) (1), prior studies report adverse drug reactions that may alter the course of the antiviral therapy. Various cutaneous and mucosal lesions may result from the use of reverse transcriptase inhibitors (RTIs), as well as protease inhibitors (2). For example, adverse skin reactions including rash, urticaria, erythema multiforme, toxic epidermolysis or Stevens-Johnson syndrome (SJS) have been associated with HAART (3). Similarly, mucosal epithelial lesions in the oral cavity include epithelial desquamation, exfoliative cheilitis, cracks, ulceration and fissure formation (4). These cutaneous and mucosal lesions may result from the use of RTIs, such as azidovudine (AZT), didanosine and efavirenz (EFV), as well as protease inhibitors (2). EFV is one of the commonly used drugs in HAART and is the first medication approved for once-daily dosing (5). Despite its antiviral efficacy at a therapeutic dose, EFV has been linked to skin lesions (3,6,7). In addition, EFV can sometimes cause severe hepatitis, central nervous system (CNS) complications, renal failure and pulmonary complications (8–11). Although these adverse effects may be viewed as hypersensitivity, direct phenotypic and genetic effects of RTIs at the cellular level have not been investigated.

Epithelial tissue regeneration can be hampered by several cell death pathways, including apoptosis, terminal differentiation, cellular senescence and autophagy. Genotoxic signals trigger premature senescence or terminal differentiation in human keratinocytes (12). Oncogene-induced senescence (OIS) is a well-characterized epigenetic phenomenon to halt cellular transformation (13). A recent report showed that mitotic arrest in OIS is mediated by autophagy, a metabolic program leading to catabolic processing of self proteins and organelles (14). Autophagic cell death is characterized by numerous autophagosomes and degradation of cytosolic proteins, whereas the nucleus remains intact until the late stage of cell death (15). This process is orchestrated by multiple protein factors, most notably the autophagy-essential proteins (ATGs), which may exceed 30 different proteins (16). Autophagy can be identified by degradation and lipidation of light chain 3 (LC3), which becomes incorporated into the membrane of autophagosomes and autophagolysosomes until it is degraded (17). Autophagy can be suppressed by chemical inhibitors of PI3K, such as 3-methylalanine (3-MA), and induced by rapamycin (18).

In the present study, we investigated the cellular effects of EFV on normal human keratinocytes (NHKs) and the underlying molecular mechanisms. Cultured NHKs exposed to EFV exhibited notable loss of cell viability accompanied by elongated morphological alterations. Assessment of the cell death pathway revealed lack of apoptotic responses but induction of autophagy in NHKs exposed to EFV. Sublethal dose of EFV rapidly induced degradation of p53. p53 degradation by EFV occurred together with a reduced mTOR level and activation of ERK phosphorylation, indicating that EFV triggers the canonical autophagic pathway in NHKs. The EFV-treated cells demonstrated premature terminal differentiation, while this effect was attenuated in cells treated with 3-MA. These data indicate that EFV limits epithelial regeneration by triggering autophagy, in part, through proteasome-dependent degradation of p53.

## Materials and methods

### Cells and cell culture

NHKs and normal human fibroblasts (NHFs) were prepared from discarded skin or mucosal tissues according to the methods described elsewhere (19). The discarded tissues were utilized to establish the primary cultures as guided by the UCLA Medical Institutional Review Board (MIRB). Human small intestine epithelial cells (CCL-241) were purchased from the American Type Culture Collection (ATCC, Manassas, VA, USA) and cultured in Hybri-Care Medium (ATCC) with 10% FBS and 30 ng/ml human EGF (Sigma, St. Louis, MO, USA). HaCaT cells represent a spontaneously immortalized human keratinocyte cell line (20), and OFK6/T cells are oral keratinocytes immortalized with the hTERT gene (21). These cells were maintained in Keratinocyte Growth Medium (Lonza, Walkersville, MD, USA). Peripheral blood mononuclear cells (PBMCs) and Jurket cells were obtained from Dr Anahid Jewett (UCLA School of Dentistry, Los Angeles, CA) and cultured in RPMI-1640 (Invitrogen) supplemented with 10% FBS. Cell viability was determined by MTT assay after a 48-h exposure to EFV according to the manufacturer's guidelines (ATCC). Replication kinetics was determined by calculating the population doubling (PD) levels according to the methods described elsewhere (19).

Organotypic reconstructs were established using NHKs (22). EFV (10 μM) was added to the culture medium at the time of airlifting for 7 days until harvested by fixing in 10% buffered formalin. Subsequently, H&E staining was performed on thick (6 μm) sagittal sections. Consecutive sections of the same specimens were used for immunohistochemistry (IHC) for proliferating cell nuclear antigen (PCNA) and K1.

### Assay of apoptotic cell death

NHKs seeded on 96-well plates were exposed to 10 μM EFV for 72 h. DNA fragmentation was determined by staining the cells and TUNEL assay using the *In Situ* Cell Death detection kit (Roche, South San Francisco, CA, USA). Apoptosis was also detected by western blotting for caspase-3 and poly-ADP-ribose polymerase (PARP) using the cells exposed to EFV and Jurkat cells exposed to ionizing radiation (IR).

### Western blotting

Whole cell extracts (50 μg) were separated on 4–20% SDS-PAGE gel and transferred onto Immobilon protein membranes (Millipore, Billerica, MA, USA). The membranes were incubated successively with the primary and the secondary antibodies. Signals were detected using ECL Western blotting detection reagents (Amersham Pharmacia Biotech, Piscataway, NJ, USA).

### Reverse transcription and real-time PCR

Total RNA was isolated from cultured cells using the RNeasy Mini kit (Qiagen, Valencia, CA, USA). cDNA was synthesized from 5 μg RNA using the Superscript first-strand synthesis system (Invitrogen). We used 1 μl cDNA for qPCR amplification using SYBR-Green I Master Mix (Roche). The primer sequences were obtained from the Universal Probe Library (Roche). PCR amplification was performed on LightCycler 480 (Roche). Second derivative Cq value determination method was used to compare fold-differences according to the manufacturer's instructions (Roche).

### Indirect in situ immunostaining

NHKs were exposed to 10 µM EFV for 48 h, fixed in 3.7% formaldehyde for 15 min, and permeabilized in 0.25% Triton X-100 in PBS for 10 min. Mouse monoclonal anti-p53 or α-tubulin and Alex Fluor^®^ 488 goat anti-mouse IgG (Invitrogen) were used as primary and secondary antibodies, respectively. Cells were then counterstained with Hoechst 33342 (3.3 μg/ml). Images were obtained using a Nikon fluorescence microscope. NHKs were treated with 10 μM EFV in the absence or presence of 5 mM 3-methylalanine (3-MA) for 48 h. Indirect immunoperoxidase staining (IPS) was performed for involucrin as described previously (23).

## Results

### EFV inhibits cell proliferation of NHKs in a cell type-specific manner

**Figure 1:**
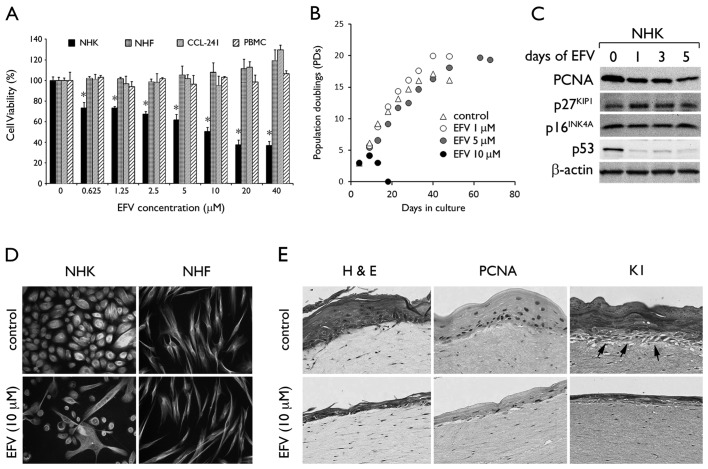
EFV exhibits cytotoxic effects on NHKs in a cell type-specific manner. (A) Cells were exposed to EFV and subjected to MTT assay after 48 h. Error bars, SD. ^*^P<0.05 against control. (B) Replication kinetics is shown in population doublings (PDs) (mean values of at least 3 different counts) against time. (C) NHKs were treated with 10 μM EFV, and western blotting was performed for PCNA, p27^KIP1^, p16^INK4A^ and p53. (D) NHKs and NHFs were treated with 10 µM EFV for 48 h. Immunofluorescence of α-tubulin is shown with Hoechst 33342. Original magnification, ×100. (E) Organotypic cultures were maintained with or without EFV (10 μM) for 7 days. H&E staining and IHC for PCNA and K1 are shown. Arrow, undifferentiated (K1-negative) basal cells. Original magnification, ×100.

To determine the effects of EFV on NHKs, we performed a cell viability assay in cultures exposed to EFV at varying doses from 0 to 40 μM. After 48 h, we found a dose-dependent reduction in cell viability with the IC_50_ at ~10 μM. As a negative control, we included 0.1% dimethylsulfoxide (DMSO). In contrast, EFV exposure conferred no cytotoxic effects on other cell types, including NHFs, PBMCs, or CCL-241 cells (Fig. 1A). We determined the long-term effects of EFV by serial subculture of primary NHKs in the presence of EFV (Fig. 1B). At 10 μM EFV, NHKs completely lost their viability within 10–15 days of exposure, while EFV at 1 and 5 μM allowed the cells to undergo continued proliferation. Notably, the cells exhibited extended lifespan at these lower doses, completing PD 20 and 19 at 1 and 5 μM EFV, respectively, while the untreated cells senescenced after PD 17. We performed western blot analysis for the proteins involved in cell cycle regulation, such as p53, p16I^NK4A^, p27^KIP1^ and PCNA in the NHKs following exposure to 10 μM EFV (Fig. 1C). After 1 day of EFV treatment, an increase in p27^KIP1^ expression and a drastic loss of p53 were noted, while the level of p16^INK4A^ did not change. PCNA expression was reduced by EFV, reflecting reduced cell proliferation. Acute exposure to 10 μM EFV led to marked changes in cellular morphology characteristic of terminally differentiated keratinocytes, while NHFs remained unchanged (Fig. 1D). We exposed the 3 dimensional (3D) organotypic culture of NHKs to EFV at 10 μM for 7 days and found epithelial atrophy, with reduced proliferating and undifferentiated basal cell content, following exposure to EFV (Fig. 1E). However, NHFs in the dermal-equivalent layer remained viable and unaltered following EFV exposure. These results demonstrate the cytotoxic and growth inhibitory effects of EFV in a cell type- and dose-dependent manner.

### EFV-induced cell death in NHKs involves autophagy but not apoptosis

**Figure 2:**
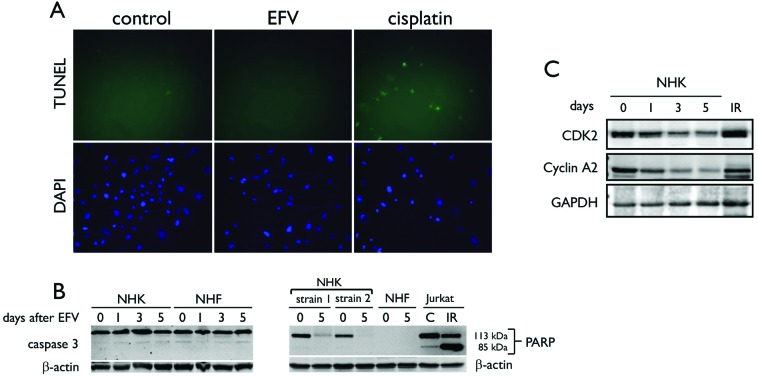
EFV does not induce apoptosis in NHKs. (A) NHKs were exposed to 10 μM EFV for 72 h and subjected to TUNEL assay to detect fragmentation of DNA ends, characteristic of apoptotic cell death. Cells treated with 10 μM cisplatin for 24 h were used as a positive control. (B) NHKs and NHFs exposed to 10 μM EFV were assayed by western blotting to detect cleavage of caspase-3 and PARP. (C) Western blotting showed progressive reduction of CDK2 and cyclin A2 in NHKs exposed to 10 μM EFV. As a control, we included Jurkat cells exposed to 5 Gy ionizing radiation (indicated by IR). β-actin and GAPDH served as loading controls.

We explored the hypothesis that apoptosis is induced by EFV in NHKs. We performed terminal deoxynucleotidyl transferase-mediated dUTP nick end-labeling (TUNEL) assay to check for DNA fragmentation in NHKs treated with 10 μM EFV for 3 days. As a comparison, we included NHKs exposed to 10 μM cisplatin, which induces apoptosis by causing DNA damage (24). Approximately 60% of the culture showed positive TUNEL staining after cisplatin treatment, while positive TUNEL staining was not detected in the control untreated cells and those exposed to EFV (Fig. 2A). EFV treatment did not cause TUNEL-positive staining in the NHKs. Western blotting was performed for detection of cleaved caspase-3 and PARP, both of which are markers of apoptotic events (25). EFV treatment did not cause caspase-3 activation or cleavage of PARP (Fig. 2B). NHKs treated with EFV showed no change in the level of caspase-3, but almost complete loss of full-length PARP. However, this was not consistent with the apoptotic response as evinced in Jurkat cells exposed to 5 Gy IR, which showed accumulation of the cleaved PARP, reflecting activation of caspase-3. Notably, EFV treatment led to a notable reduction in the levels of CDK2 and cyclin A2 (Fig. 2C), which are required for entry into mitosis (26), suggesting potential arrest of the cell cycle in the S phase. Hence, the cell death noted in NHKs following exposure to EFV did not involve apoptosis.

**Figure 3:**
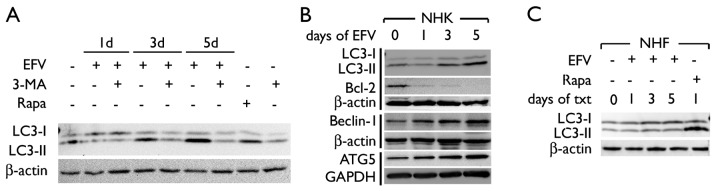
EFV induces autophagy in NHKs. (A) NHKs were exposed to 10 µM EFV with or without 3-MA. Levels of LC3-I and LC3-II were determined by western blotting. (B) Levels of LC3-I, LC3-II, Bcl-2, Beclin-1 and ATG5 were determined by western blotting. (C) NHFs exposed to 10 μM EFV were assayed for the levels of LC3-I and LC3-II by western blotting. The cells treated with rapamycin (100 nM) (Rapa) were used as a positive control. β-actin or GAPDH served as a loading control. Days of txt, days of drug treatment.

Next, we examined the occurrence of autophagy, known as an alternative pathway of programmed cell death (27). We assessed the levels of LC3-I and LC3-II, which represent the parent form and the cleaved form, respectively. Following 3 days of EFV treatment, we noted an increased level of LC3-II, while this was abolished by co-treatment with 3-MA (Fig. 3A). As a control, NHKs were exposed to rapamycin, which induces the level of LC3-II. EFV treatment almost completely abolished the level of Bcl-2 by day 1. There was a time-dependent increase in the levels of Beclin-1 and ATG5, and this paralleled the increase in LC3-II in the NHKs following treatment with EFV (Fig. 3B). The LC3-II level did not change in the NHFs exposed to EFV, whereas rapamycin led to a notable induction in LC3-II (Fig. 3C). These data suggest that EFV induces autophagy in NHKs but not in NHFs, and this may explain the cell type-specific cytotoxicity of EFV.

### EFV stimulates proteosome-dependent degradation of p53, loss of mTOR, and activation of ERK1/2 in NHKs

**Figure 4:**
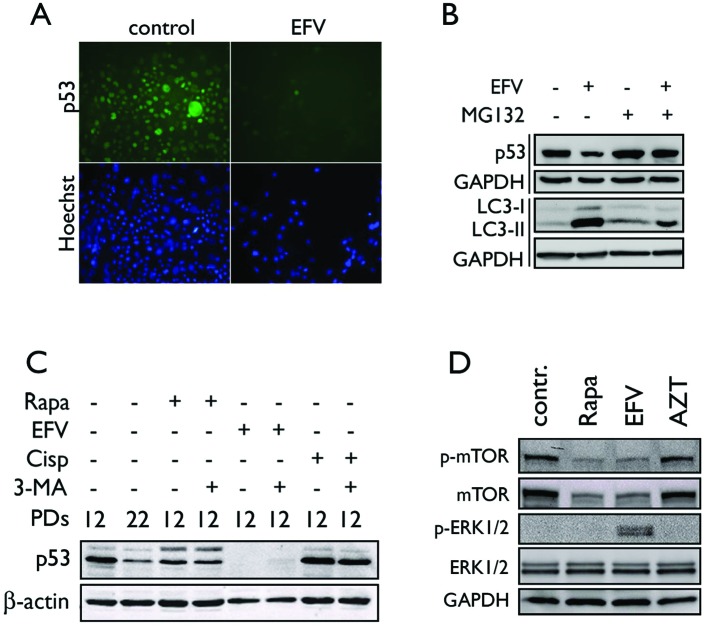
EFV causes proteosome-dependent degradation of p53. (A) NHKs exposed to 10 μM EFV for 48 h were stained for p53 by indirect immunofluorescence staining and Hoechst 33342 counterstain. Original magnification, ×100. (B) NHKs were exposed to 10 μM EFV for 48 h with or without the addition of 10 μM MG132 2 h prior to collection. Western blotting was performed for p53 and LC3. (C) NHKs at PD 12 were exposed to 10 μM EFV for 48 h, 100 nM rapamycin (Rapa) for 5 days or 10 μM cisplatin (Cisp) for 24 h in the absence and presence of 3-MA (5 mM). The p53 level was determined by western blotting. (D) NHKs were exposed to EFV, rapamycin (Rapa), or AZT, and the cells were assayed for phosphorylated and total mTOR, and phosphorylated and total ERK1/2. β-actin or GAPDH served as a loading control.

Previous studies have demonstrated that cytoplasmic p53 plays a causative role in autophagy (28). Western blotting showed that EFV treatment led to a rapid reduction in p53 in NHKs (Fig. 4B). This was confirmed by immunofluorescence staining of p53, which showed loss of nuclear and cytoplasmic p53 in the EFV-treated cells (Fig. 4A). Since p53 undergoes ubiquitin-dependent proteasomal degradation, we exposed the cells to EFV in the presence of MG132, a proteasome inhibitor. EFV treatment led to a reduction in the p53 level and a strong increase in LC3-II accumulation, reflecting induction of autophagy (Fig. 4B). MG132 blocked the EFV-mediated p53 degradation and notably reduced the level of LC3-II in the cells exposed to EFV. On the contrary, co-treatment of 3-MA, which suppressed EFV-mediated autophagy in NHKs, did not inhibit p53 degradation in the cells exposed to EFV (Fig. 4C), suggesting that p53 degradation does not result from EFV-mediated autophagy. The EFV-treated NHKs showed reduced phosphorylation of mTOR (Ser2448), similar to the cells treated with rapamycin (Fig. 4D). This occurred together with a reduced protein level of mTOR. In contrast, the azidothymidine (AZT)-treated cells exhibited no changes in mTOR phosphorylation. EFV treatment also led to phosphorylation of ERK1/2 (Thr202 and Tyr204) while other treatments showed no effect. The above data suggest that EFV-mediated autophagy in NHKs occurs with loss of p53 and mTOR, and activation of the ERK pathway.

### Autophagy induced by EFV is linked with terminal differentiation of NHKs

**Figure 5:**
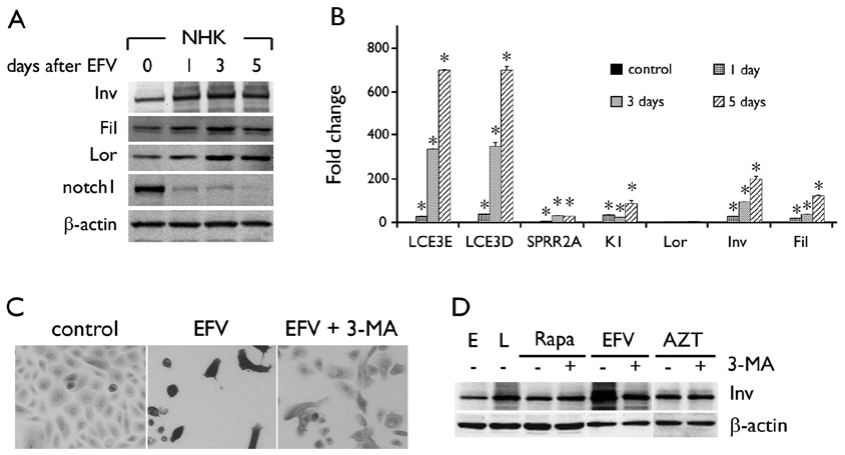
EFV induces terminal differentiation in NHKs. (A) Western blotting was performed with EFV-treated NHKs to detect various proteins as indicated. (B) mRNA expression levels for differentiation-associated genes were determined by qPCR. Error bars, SD. ^*^P<0.05 against control. (C) NHKs were exposed to 10 μM EFV for 48 h with or without 3-MA (5 mM) and stained for involucrin by IPS. Original magnification, ×100. (D) Involucrin expression was determined in NHKs exposed to rapamycin (Rapa), EFV or AZT in the absence or presence of 3-MA. E, proliferating cells at PD 12; L, senescent cells at PD 22 were included as controls. β-actin served as a loading control. Inv, involucrin; Fil, filaggrin; Lor, loricrin, K1, keratin 1.

Prior to cell death, the EFV-treated cells exhibited morphological changes resembling terminal differentiation (Fig. 1D). This was confirmed by western blotting for the markers of keratinocyte differentiation, i.e. involucrin, filaggrin and loricrin. The levels of these markers increased in NHKs exposed to EFV in a time-dependent manner (Fig. 5A). After 5 days of EFV exposure, involucrin expression was increased to a level similar to calcium treatment (data not shown), which triggers terminal differentiation (19). EFV-induced keratinocyte differentiation occurred with loss of the Notch1 protein level after exposure to EFV. We confirmed the induction of the genes involved in keratinocyte differentiation by qPCR. All tested genes, LCE3E, LCE3D, SPRR2A, keratin 1, loricrin, involucrin and filaggrin, were progressively induced at the mRNA expression level by EFV treatment (Fig. 5B). *In situ* immunoperoxidase staining revealed the increased intensity of involucrin staining and lack of cell proliferation in NHKs exposed to EFV (Fig. 5C). However, when the cells were co-treated with EFV and 3-MA, there was a reduction in involucrin staining and partial recovery of replicating and surviving cells. Western blotting revealed that EFV strongly induced the involucrin level beyond that of senescent NHKs at late-passage (PD 22), and 3-MA treatment notably reduced the involucrin protein level in the EFV-treated cells (Fig. 5D). Notably, rapamycin or AZT did not trigger keratinocyte differentiation. Thus, EFV-mediated autophagy in the NHKs was uniquely linked with aberrant keratinocyte differentiation, which is partially responsible for the cytotoxic effects of the drug.

## Discussion

Exposure of NHKs to EFV led to autophagic cell death associated with terminal differentiation. This was demonstrated by rapid loss of cell proliferation and viability that accompanied induction of LC3-II and the markers of keratinocyte terminal differentiation in cells exposed to EFV. Organotypic culture study showed that EFV treatment eliminates the undifferentiated and proliferating cells in the epithelium. This leads to lack of epithelial regeneration and causes atrophy. Therefore, aberrant differentiation of keratinocytes caused by EFV would, at least in part, be responsible for cutaneous adverse effects noted in patients. These phenotypic responses occurred uniquely in NHKs but not in other cell types, i.e. fibroblasts, intestinal cells or leukocytes. The 3D tissue reconstruction model revealed epithelial atrophy and the lack of basal cell proliferation and tissue regeneration upon exposure to EFV, while NHFs in the dermal equivalent layer remained unchanged. This cell type-specificity was paralleled by induction of autophagy in NHKs but not in NHFs, indicating that the cytotoxicity of EFV is linked with autophagy. On the contrary, induction of apoptosis was not noted in EFV-treated cells. We also tested several other genotoxic agents, such as actinomycin D, dexamethasone, etoposide and methyl-nitro-nitrosoguanine (data not shown). Apoptotic signaling from these strong genotoxic agents was extremely weak or absent in NHKs. Likewise, we recently reported that IR failed to trigger apoptosis in NHKs, while it strongly induced apoptosis in Jurkat cells (29). Thus, apoptosis was poorly induced in these cells.

EFV treatment increased the protein level of Beclin-1, ATG5 and p27^KIP1^, and drastically reduced p53 and Bcl-2. The increased p27^KIP1^ level may have resulted from autophagy through the AMP-activated protein kinase pathway (30). Loss of Bcl-2 may allow Beclin-1 binding to hVps34, a class III PI3K (31), thereby promoting autophagy in the cells treated with EFV. EFV also led to marked reduction in cyclin A2, which is required for mitotic entry (26), suggesting possible S phase arrest in the treated cells. Loss of cyclin A2 may have contributed to the growth inhibitory effects of EFV on NHKs. Autophagy occurs along with proteosome-dependent degradation of p53; specific inhibition of p53 is sufficient to trigger autophagic cell death (28). p53 degradation in the EFV-treated NHKs occurred quite rapidly in a proteosome-dependent manner, and blockage of the p53 degradation notably reduced the autophagic response to EFV. p53 degradation did not occur in NHFs, which showed no phenotypic changes to EFV (data not shown). We previously showed that the levels of PCNA, involucrin, p16^INK4A^ and p53 do not change in exponentially replicating cells until the cells approach replicative senescence (32,33). In the present study, the inhibitory effect of EFV was noted primarily on p53, while that of p16^INK4A^ was not evident (Fig. 1C). In addition, we used rapidly proliferating cells for the experiments. Thus, the possibility that the loss of p53 occurred through replicative senescence during EFV exposure is very remote. These data suggest that p53 degradation in NHKs may have a causative role in autophagy in response to EFV.

Autophagy is often linked with a variety of other cellular processes, such as senescence, ER stress, or differentiation. A recent study showed that autophagy mediates the mitotic arrest during OIS and demonstrated the interdependence between the two processes (14). Our data showed that autophagy was linked to terminal differentiation in NHKs as a mechanism of cell death. The EFV-treated NHKs demonstrated elongated morphology in culture and strongly expressed the markers of keratinocyte differentiation. These phenotypic changes including cell death were partially inhibited by 3-MA, an inhibitor of the PI3K pathway (18). Rapamycin also induced autophagy in NHKs but did not trigger differentiation, suggesting that the mechanisms causing autophagy were different between rapamycin and EFV. Keratinocyte differentiation is regulated by the notch signaling pathway (34). However, EFV-induced differentiation occurred without notch induction. Rather, the notch protein level rapidly decreased in cells exposed to EFV, presumably due to p53 degradation since p53 is required for notch expression (34).

Although autophagy is a method of programmed cell death and can function as a tumor-suppressive mechanism, it may also lead to cell survival under stressful conditions. Beclin-1 was found to be deleted in large portions (50–75%) of various types of human cancers, including breast and ovarian (35,36). Introduction of beclin-1 into a cancer cell line led to autophagy and loss of cell proliferation and *in vivo* tumorigenicity (37). A subsequent study showed that monoallelic deletion of beclin-1 in a mouse model led to increased tumorigenesis associated with reduced autophagy (38). These studies suggest the tumor suppressive effects of autophagy. In contrast, autophagy does play a role in maintaining the viability of highly proliferative cancer cells, particularly in the center of the tumor mass where the cells are under severe metabolic stress. A study by Degenhardt *et al*(39) demonstrated induction of autophagy in regions of metabolic stress to mitigate the ischemic cell death. Furthermore, autophagy was found to be induced in leukemic cells undergoing anti-cancer therapy with a histone deacetylase inhibitor, such as suberoylanilide hydroxamic acid (SAHA), as a protective and survival mechanism (40). Chemical and genetic disruption of autophagy led to enhanced anticancer efficacy of SAHA in the present study. Therefore, autophagy may indeed be a mechanism by which cancer cells gain resistance to chemotherapeutic agents or protect cancer cells from metabolic stress.

In HIV^+^ patients undergoing long-term therapeutic exposure to EFV, the occurrence of autophagy and its contribution to tumorigenesis need to be investigated. The mean plasma concentration of EFV in HIV^+^ patients under the antiretroviral regimen was found to be 8.7 μM for those who responded to therapy (41). Another study showed the mean plasma concentration to be 6.9 μM with a wide range from 0.4 to 48 μM, and suggested an effective therapeutic plasma concentration of EFV at 3–13 μM (42). Thus, 10 μM EFV used in the present study was within the clinically relevant concentration at which EFV exhibits an antiviral effect. It is possible that EFV-induced autophagy has contrasting effects on cells at different concentrations. As shown in Fig. 1D, EFV caused cell death at 10 μM but allowed enhanced cell proliferation at 1 and 5 μM, extending the replicative lifespan of cells. We also found that chronic exposure of immortalized oral keratinocytes harboring the human papillomavirus (HPV) genome to 5 μM EFV interfered with terminal differentiation and led to a transformed phenotype (data not shown). Although EFV triggered terminal differentiation and autophagic cell death in NHKs at 10 μM, it may have a tumor-promoting effect at a lower concentration by protecting aberrant cells from metabolic stress and suppressing the cell death pathway. These possibilities warrant further investigation.
